# Effects and safety of tanreqing injection on viral pneumonia: A protocol for systematic review and meta-analysis

**DOI:** 10.1097/MD.0000000000021808

**Published:** 2020-08-28

**Authors:** Hui Liu, Xue-Fei Ding, Rui Guo, Meng-Fan Zhao, Di Deng, Yu Hao, Yi Wang

**Affiliations:** a Department of Immunology and Microbiology, School of Life Science, Beijing University of Chinese Medicine; b Department of Emergency, Beijing Shunyi Hospital of Traditional Chinese Medicine; c Experimental Research Center, China Academy of Chinese Medical Sciences, Beijing, China.

**Keywords:** effect, safety, tanreqing injection, viral pneumonia

## Abstract

**Background:**

Viral pneumonia is a common respiratory disease that leads to high mortality around the world. Tanreqing (TRQ) injection has been widely used to treat viral pneumonia in China. However, the efficiency and safety of TRQ injection for viral pneumonia have not been scientifically and methodically evaluated up to now. Thus, this protocol describes a plan of performing a systematic review and meta-analysis to evaluate the efficacy and safety of TRQ injection on patients with viral pneumonia.

**Methods:**

Only randomized controlled trials will be enrolled in our study, and we will search eligible studies in the following electronic databases: PubMed, Embase, Cochrane Central Register of Controlled Trials, Clinical Trials, China National Knowledge Infrastructure, the Wanfang database, the Chinese Scientific Journal Database, and the Sinomed. The total effective rate of clinical efficacy will be used as primary outcome. Time to relieve symptoms, incidence of adverse reactions, and the laboratory parameters will be used as secondary outcomes. Any side effects and adverse events will be recorded and assessed as safety outcomes. Study inclusion, data extraction, and quality assessment will be performed independently by 2 reviewers, and any disagreement will be resolved by a third reviewer. After that, data synthesis and subgroup analysis will be conducted with the Review Manager V.5.3.3 software.

**Results:**

This review will provide a high-quality synthesis to assess the effectiveness and safety of TRQ injection for viral pneumonia patients.

**Conclusion:**

Our study will provide comprehensive evidence to decide whether TRQ injection is effective and safe for viral pneumonia patients.

**Prospero registration number:**

PROSPERO CRD42020164164

## 1 Introduction

Pneumonia is a common disease that remains the leading killer of elderly people in developed countries and young children in developing countries, resulting in heavy burden in the world.^[1,2]^ In the recent prospective pneumonia etiology studies, respiratory viruses were more commonly detected than bacteria in both adults and children, accounting for greater than 25% of detections in adults and greater than 70% in children.^[3]^ The most commonly detected respiratory viruses are human rhinoviruses, influenza viruses, parainfluenza viruses, adenoviruses, human metapneumovirus, coronaviruses, and respiratory syncytial virus.^[4]^

The primary symptoms of viral pneumonia are fever, cough with or without phlegm, shortage of breath, and pharyngalgia. If uncontrolled well, respiratory distress syndrome, and shock may be caused secondarily to these symptoms and even cause deaths. However, the pathogenesis of viral pneumonia has not been clear enough, which vastly limits the clinical treatment at present. The current treatment for viral pneumonia is mainly supportive, and the neuraminidase inhibitors oseltamivir and peramivir are the Food and Drug Administration-approved antiviral drugs for the viral pneumonia treatment in adults.^[5]^ However, efficacy of antiviral drugs is usually reduced by the high degree of variability of respiratory virus and the emergence of drug-resistant strains as well as drug adverse reactions.

Traditional Chinese Medicine (TCM) has been shown its unique advantages in treating respiratory diseases for thousands of years, which is characterized by multi-link, multi-target, and minimal side-effects.^[6–10]^ Tanreqing (TRQ) injection is one of the important TCM injection, which includes extracts from *Flos Lonicerae (Lonicera japonica Thunb*), *Radix Scutellariae (Scutellaria baicalensis Georgi), Fructus Forsythiae (Forsythia suspensa Thunb), Fel ursi, (Fel Ursi) and Cornu saigae tataricae (Naemorhedus goral Hard-wicke*),^[11]^ has marked effects of clearing heat, reducing inflammation, detoxification and alleviating cough.^[12]^ Except for those functions in TCM theory, modern pharmacologic studies confirmed that

TRQ injection can protect against various viruses, such as adenovirus, syncytial virus, influenza virus, and coxsackie virus, and can also relieve airway inflammation and excessive mucus caused by virus.^[13]^ Thus, TRQ injection is widely used to treat the upper respiratory tract infections, acute pneumonia, acute bronchitis, and many clinical trials reported that TRQ injection could significantly alleviate symptoms and improve signs.^[14,15]^

So far, there have been several systematic reviews and meta-analysis about TRQ for community acquired pneumonia, chronic obstructive pulmonary diseases, and tuberculosis,^[14,16,17]^ but none was specifically for viral pneumonia. Besides, according to clinical reports, TRQ has some side effects in clinical applications, so that whether it is safe enough is still controversial. Therefore, the aim of this systematic review is to assess the efficacy and safety of TRQ injection on Viral Pneumonia.

## 2 Objectives

The objective of this study is to analyze various randomized controlled trials (RCTs) and provide evidence for the effectiveness and safety of TRQ injection on viral pneumonia.

## 3 Methods

The methodology of this systematic review will be guided by the Preferred Reporting Items for Systematic Reviews and Meta-Analyses statement.^[18]^

### 3.1 Inclusion criteria

#### 3.1.1 Types of studies

All the RCTs of TRQ injection for the treatment of viral pneumonia patients will be included. Meanwhile, case reports, retrospective studies, and nonclinical studies will be excluded.

#### 3.1.2 Types of participants

We will include studies on patients of any age, race, gender, severity, and duration of disease, who are diagnosed with viral pneumonia. Participants with critical organ dysfunction or severe mental disorders will be excluded. Additionally, patients with atopy will be excluded. The specific criteria are as follows:

##### 3.1.2.1 Inclusion criteria

(1) Diagnosed with viral pneumonia.
(2) The age, race, gender, severity, and duration of disease are not limited.
(3) Informed consents were got from patients themselves or their families.

##### 3.1.2.2 Exclusion criteria

(1) Critical organ dysfunction, such as severe cardiopulmonary dysfunction, hepatic or kidney failure, or serious uncontrolled infection.
(2) Severe mental disorders or any other diseases influence compliance.
(3) Allergic condition.

#### 3.1.3 Types of Interventions

Patients in both treatment and control group undergo basic symptomatic treatment such as pyretolysis, alleviating cough, dissolving sputum, and antiasthma. In the treatment group, the intervention will be TRQ injection or TRQ injection plus western medicine (antibiotics or antiviral drugs). Under normal conditions, patients in control group will be treated with western medicine (antibiotics or antiviral drugs).

#### 3.1.4 Types of outcome measures

##### 3.1.4.1 Primary outcomes

The primary outcome will be the total effective rate of clinical efficacy (number of people curative and effective/total number of people treated).

##### 3.1.4.2 Secondary outcomes

The additional outcomes will be as follows:

(1) Chest radiograph normalization rate;
(2) Time to relieve symptoms like fever, cough, crackles, and absorption of shadows on X-ray.
(3) Laboratory parameters and etiological examination, such as peripheral blood white blood cells and neutrophil count, the levels of C Reactive Protein (CRP) and procalcitonin, the levels of viral antigens and viral antibodies in respiratory or serum samples, and so on.

All reported side effects and adverse events will be included as safety outcomes. The common side effects of TRQ injection, such as anaphylaxis, drug eruption, nausea and vomiting, arrhythmia, haematuria, and so on. will also be recorded and evaluated respectively.

### 3.2 Search methods

#### 3.2.1 Electronic searches

An electronic search strategy will be carried out in the following databases including the PubMed, Embase, Cochrane Central Register of Controlled Trials, Clinical Trials, China National Knowledge Infrastructure, the Wanfang database, the Chinese Scientific Journal Database, and the Sinomed. The search duration will be from inception to December 31th of 2019. In addition, we will search the reference lists of the included studies, ongoing trials and unpublished conference proceedings, which will be identified using the Current Controlled Trials (http://www.controlled-trials.com) and WHO International Clinical Trials Registry Platform (http://www.who.int/trialsearch) for any unpublished or relevant ongoing trials.

#### 3.2.2 Search strategy

The following terms will be used for the search:

(1) RCT
(2) Controlled clinical trial
(3) Randomized
(4) Randomly
(5) Placebo
(6) Trial
(7) 1 or 2–6
(8) Viral Pneumonia
(9) Viral Pneumonias
(10) Pneumonias, Viral
(11) Pneumonia, Viral
(12) Or/8-11
(13) TRQ
(14) TRQ injection
(15) TRQ
(16) TRQ injection
(17) Or/13-16
(18) 7 and 12 and 17

### 3.3 Data collection and analysis

#### 3.3.1 Selection of studies

Two investigators will independently screen the titles and abstracts of all included studies according to the established inclusion and exclusion criteria. Then, they will read the full text of potentially eligible studies to confirm the final included studies.

Any disagreements on study selection will be resolved by consensus or a third reviewer. We will contact either the first authors or the corresponding authors of the included studies if there are issues to be clarified.

#### 3.3.2 Data extraction and management

All required data will be extracted by 2 investigators independently, and a standard extraction form will be established via Excel to extract substantial contents. Disagreements will be solved by a third investigator through discussion. Data in detail will be extracted from each study as follows: title, the author, the year of publication, journal, country, participants, study design, sample size, randomization, characteristics of the interventions, allocation concealment, incomplete data, blinding, outcomes, selective reporting, adverse events, and follow-up, sources of funds and conflicts of interest. We will contact the authors when relevant data is missing.

#### 3.3.3 Assessment of the risk of bias

The risk of bias of each included article will be evaluated by 2 independent reviewers according to the Cochrane Handbook of Systematic Reviews of Interventions.^[19]^

We will assess the risk of bias from the following domains:

(1) selection bias, including generation of random sequence and allocation concealment.
(2) performance bias, including blinding of participants and personnel.
(3) detection bias, including blinding of outcome assessment and the blinding of the data assessor.
(4) attrition bias, which refers to incomplete outcome data.
(5) reporting bias, including selective reporting, whether or not all data are reported.
(6) other bias, such as conflicts of interest.

We will classify risk of bias into 3 levels: “high risk,” “low risk,” and “unclear risk,” and the disagreement will be resolved through discussion or consult the third reviewer.

#### 3.3.4 Measurements

Dichotomous data and continuous data will be included in the outcomes of the studies. For dichotomous data, we will express the results as risk ratio with 95% confidence intervals. For continuous data, standardized mean difference or mean difference with 95% confidence intervals will be used to summarize the results.

#### 3.3.5 Management of missing data

If any data information is insufficient of included studies, we will contact either the first or corresponding author via telephone or email to request the missing data. We will analyze the available data to accomplish our analysis and explain the potential impact of missing data if we still cannot get the accurate data.

#### 3.3.6 Assessment of heterogeneity

We will assess heterogeneity between the included studies by Chi-squared test (*χ*^2^ test) and *I*^2^ test according to the guidelines in Cochrane Handbook. If *I*^*2*^ < 50% or *P* > .1, which indicates that there is no statistical heterogeneity, and then a fixed effects model will be adopted. While, If *I*^2^ ≥ 50% or *P* < .1, indicating the heterogeneity among studies are statistically significant, and a random-effect model will be employed.

#### 3.3.7 Assessment of reporting biases

If there are no less than 10 RCTs included in the meta-analysis, we will examine the potential report biases by generating funnel plots. Otherwise, Quantitative analysis will be performed by Egger test.^[20]^

#### 3.3.8 Data synthesis

Meta-analysis will be carried out using Review Manager (V.5.3) statistical software provided by Cochrane Collaboration. We will carry out the fixed effect model to meta-analysis if there is no obvious heterogeneity among the trials, and a random effect model will be adopted if there is significant statistical heterogeneity.

#### 3.3.9 Subgroup analysis

If a sufficient number of random trials and the evident heterogeneity are identified, we will perform subgroup analyses according to the following items:

(1) Viral pneumonia in different ages, such as children, adults, and the elderly;
(2) The efficacy of TRQ alone versus western medicine or combined with western medicine vs western medicine;
(3) Severity of viral pneumonia, such as the mild, moderate, and severe condition.

#### 3.3.10 Sensitivity analysis

Sensitivity analysis will be performed to evaluate the robustness and stability of pooled outcomes after removing the low-quality studies. The final results will depend on the missing data, risk of bias, sample size, and quality of methods of each study.

#### 3.3.11 Grading the quality of evidence

The Grading of Recommendations Assessment, Development, and Evaluation guidelines will be used to evaluate the quality of evidence.^[21]^ Levels of evidence quality will be classified into 4 levels: high, moderate, low, or very low.

#### 3.3.12 Ethics and dissemination

Since our study will not include animals or individuals, it is not necessary for ethical approval. The results of this review will be published in related conferences or peer-reviewed journals.

## 4 Discussion

As a traditional Chinese herbal medicine injection, TRQ injection has been approved by the China Food and Drug Administration and widely used for respiratory infections to alleviate symptoms and improve signs in clinics. Although many clinical studies confirmed that TRQ injection has a significant effect on viral pneumonia, there is no comprehensive systematic review offering enough evidence for such treatment. Thus, in this study, we will integrate and analyze the most comprehensive clinical studies to investigate the efficacy and safety of TRQ injection on viral pneumonia. At the same time, the study has been registered on PROSPERO, making it more transparent and trustworthy. We hope that this finding may provide evidence and instructions for the application of TRQ injection treatment for viral pneumonia. Of course, there are some limitations in this review. For example, we will only search English and Chinese databases. Additionally, different age of the patients and different severity of disease may run the risk of heterogeneity, subgroup analyses will be needed for evaluation accuracy.

## Author contributions

**Conceptualization:** Hui Liu, Xuefei Ding.

**Methodology:** Hui Liu, Xuefei Ding, Rui Guo, Di Deng.

**Project administration:** Hui Liu, Xue-Fei Ding, Meng-Fan Zhao.

**Supervision:** Yu Hao, Yi Wang.

**Writing – original draft:** Hui Liu, Xue-Fei Ding.

**Writing – review & editing:** Yu Hao, Yi Wang.
